# Gonadectomy in Mito-Ob mice revealed a sex-dimorphic relationship between prohibitin and sex steroids in adipose tissue biology and glucose homeostasis

**DOI:** 10.1186/s13293-018-0196-4

**Published:** 2018-08-29

**Authors:** Yang Xin Zi Xu, Sudharsana Rao Ande, Suresh Mishra

**Affiliations:** 1 Department of Physiology and Pathophysiology, Faculty of Health Sciences, University of Manitoba, Rm. 843 JBRC/715 McDermot Avenue, Winnipeg, MB R3E 3P4 Canada; 2 Department of Internal Medicine, Faculty of Health Sciences, University of Manitoba, Rm. 843 JBRC/715 McDermot Avenue, Winnipeg, MB R3E 3P4 Canada

**Keywords:** Prohibitin, Obesity, Adipocyte differentiation, 17β-estradiol, Testosterone, Sex differences, Transgenic mice

## Abstract

**Background:**

Recently, we have developed a novel transgenic mouse model by overexpressing prohibitin (PHB) in adipocytes, which developed obesity due to upregulation of mitochondrial biogenesis in adipocytes, hence named “Mito-Ob.” Interestingly, only male Mito-Ob mice developed obesity-related impaired glucose homeostasis and insulin sensitivity, whereas female Mito-Ob mice did not. The observed sex differences in metabolic dysregulation suggest a potential involvement of sex steroids. Thus, the main aim of this study is to investigate the role of sex steroids on the overall phenotype of Mito-Ob mice through gonadectomy, as well as direct effect of sex steroids on adipocytes from Mito-Ob mice in vitro.

**Methods:**

Mito-Ob mice and wild-type CD-1 mice were gonadectomized at 12 weeks of age. Age- and sex-matched sham-operated mice were used as controls. Body weight, white adipose tissue, glucose tolerance, and insulin sensitivity were analyzed 3 months post-surgery. Differentiation of adipocytes isolated from female and male Mito-Ob mice were studied with and without sex steroids.

**Results:**

Gonadectomy significantly reduced body weight in Mito-Ob mice compared with sham-operated mice, whereas the opposite trend was observed in wild-type mice. These changes occurred independent of food intake. A corresponding decrease in adipose tissue weight was found in gonadectomized Mito-Ob mice, but depot-specific differences were observed in male and female. Gonadectomy improved glucose tolerance in male wild-type and Mito-Ob mice, but the effect was more pronounced in wild-type mice. Gonadectomy did not alter insulin sensitivity in male Mito-Ob mice, but it was improved in male wild-type mice. In primary cell cultures, testosterone inhibited adipocyte differentiation to a lesser extent in male Mito-Ob preadipocytes compared with the wild-type mice. On the other hand, preadipocytes from female wild-type mice showed better differentiation potential than those from female Mito-Ob mice in the presence of 17β-estradiol.

**Conclusions:**

PHB requires sex steroids for the development of obese phenotype in Mito-Ob mice, which differentially affect glucose homeostasis and insulin sensitivity in male and female. It appears that PHB plays sex- and adipose depot-specific roles and involves additional factors. In vitro studies suggested that PHB differently influenced adipocyte differentiation in the presence and absence of sex steroids. Overall, this study along with available information in the literature indicated that a multifaceted relationship exists between PHB and sex steroids, which may work in a cell/tissue type- and sex-specific manner.

## Background

Sex differences have profound effects on the susceptibility and pathophysiology of obesity-related diseases and their complications [1]. One of the sex-biasing factors is the effect of gonadal sex steroids. Estradiol and testosterone play important roles in metabolism including energy balance and fat distribution in the body. Men with obesity are shown to exhibit a progressive decline of testosterone level and increased body weight [2]. In women, epidemiological data suggest that obesity and metabolic dysregulation are more prevalent after menopause, and estrogens have been used as a therapeutic agent to reverse these complications [3–5]. At the molecular level, sex steroids regulate gene expression through binding to their respective receptors expressed in adipose tissues [6–8]. However, there is a lack of knowledge on downstream mediators involved in the actions of sex steroids in regulating adipogenesis, and critical factors that contribute to sex differences in adipose tissue biology and pathobiology remain elusive.

Recent studies have highlighted the potential relevance of mitochondria in the cellular physiology of adipocytes and its impact on systemic metabolic regulation [9, 10]. Adipocytes interpret nutritional and hormonal cues in their microenvironment and coordinate mitochondrial response either to oxidize incoming fatty acid and carbohydrate or to store them in the form of triacylglycerol until signal for release. Of note, the effect of male and female sex steroids on mitochondria is not exactly the same. However, it is not known whether such differences contribute to sex differences in adipose tissue functions.

We discovered that prohibitin (PHB), which is known to function in mitochondrial biology, has an important role in adipocyte differentiation in vitro [11]. This finding is further confirmed by similar findings by others [12, 13]. To explore the role of PHB in adipose tissue biology at the systemic level, we have developed a transgenic mouse model by overexpressing PHB in adipocytes under the fatty acid-binding protein-4 (*Fabp4*, also known as adipocyte protein-2 (*aP2*) gene promoter) [14]. Phenotypic characterization of PHB transgenic mice revealed that they developed obesity due to upregulation of mitochondrial biogenesis in adipocytes independent of diet, hence the name Mito-Ob mice [14]. While they developed obesity in a sex-neutral manner, the progression of obesity-related impaired glucose homeostasis and insulin sensitivity was male-specific [14]. Furthermore, serum triglyceride and cholesterol levels were significantly lower in female Mito-Ob mice, whereas free fatty acid level was significantly higher in male Mito-Ob mice [14]. In addition, adiponectin levels were significantly higher in female Mito-Ob mice, while leptin levels were higher in male Mito-Ob mice [14]. Collectively, these findings indicated a sex-dimorphic role of PHB in adipocytes and adipose tissue biology. Of note, Mito-Ob mice started to gain weight during puberty, indicating a relationship between gonadal sex steroids and the observed phenotypes. Emerging evidence from other laboratories also supported a role of PHB in modulating sex steroid actions in a variety of cells and tissues, as well as PHB as a downstream target of sex steroids [15–17]. Specifically, the effect of sex steroids on adipocytes, the site of PHB overexpression in Mito-Ob mice, may contribute to the sex-dimorphic metabolic characteristics described. Based on these findings, the main aims of this study are to investigate (1) the role of sex steroids on adipose tissue biology and glucose homeostasis in the Mito-Ob mouse model through gonadectomy and (2) the direct effect of sex steroids on the differentiation of preadipocytes isolated from Mito-Ob mice in vitro.

## Methods

### Animal care and body weight

Male and female Mito-Ob mice and their wild-type CD-1 counterparts were caged separately in groups of 4, and allowed normal chow (Mouse Diet 5015; Catalog # 0001328; LabDiet, St. Louis, MO) and water ad libitum. This diet is a complete life cycle diet formulated using managed formulation, delivering Constant Nutrition® (% calories provided by protein (19.752), fat (26.101), and carbohydrates (54.148)). This formulation contains 11% fat to fulfill the metabolic needs in maintaining maximum reproduction for postpartum mating where females are under simultaneous stress of lactation and gestation. Body weight was recorded weekly after weaning, and food intake was recorded up to 6 months of age. All experiments involving animals were carried out in accordance with the Canadian Council on Animal Care requirements and as per Animal Use Protocol #16-005 approved by the Animal Care and Use Committee, University of Manitoba.

### Gonadectomy

Mito-Ob and wild-type mice were each distributed into experimental groups of eight animals per group as follows: (1) female sham-operated, (2) female ovariectomized, (3) male sham-operated, and (4) male orchiectomized. Mice were gonadectomized at 12 weeks of age. Weights of animals were recorded before surgery. After giving isoflurane as anesthesia, gonadal area was cleaned with chlorohexanol followed by 70% alcohol, and Metacam (2 mg/kg) was injected subcutaneously as analgesics.

For orchiectomy, male mouse was placed in ventral recumbency and a 1-cm median incision was made in the scrotum, and the skin was retracted to expose the tunica. The tunica was then pierced to extract testes one at a time. The testes were raised to expose the underlying spermatic cord, which was clamped and ligated at the confluence of blood vessels and epididymis. In sham-operated mice, the same procedures were performed except that testes were not removed. Skin incision was sutured up with adhesive wound clips.

For ovariectomy, female mouse was placed in ventral recumbency, and a 1-2-cm skin incision was made at the dorsal midline halfway between the caudal end of the ribcage and the base of the tail. The fascia was cleared away using blunt-end dissection, and the underlying muscle wall was pierced on both sides 1 cm lateral to the spine. Ovary and oviduct were exteriorized through the muscle wall. A hemostat was clamped around the uterine vasculature between the oviduct and uterus. Each ovary and part of oviduct were removed with a single cut. In sham-operated mice, the same procedures were performed except that ovaries were not removed. Skin incision was sutured up with adhesive wound clips.

After the surgery, each mouse was given its own cage to recover. Postoperative health card was used to record any abnormalities including signs of discomfort and pain. Mice were monitored every hour during the first 5 h after emergence from anesthesia, and twice a day after for 3 days. 1 mg/kg of Metacam was given daily for 3 days. After a week, would clips were removed. Body weight was taken thereafter every week up to 6 months of age.

### Tissue histology

Adipose tissues from 6-month-old Mito-Ob and wild-type mice were collected and weighed. Tissue samples were fixed in 10% formalin in phosphate-buffered saline (PBS), dehydrated, and embedded in paraffin according to routine histologic method for microscopy [18]. Each slide contained a 5-μm tissue section stained with hematoxylin and eosin.

### Adipocyte quantification

Histology sections were viewed under a light microscope at 10× and 40× magnifications, and five images were randomly generated at each magnification using Infinity-2 digital camera (Lumenera Corporation, Ottawa, ON, Canada). All 40× images were analyzed with Adiposoft-ImageJ [19]. First, the image was split into red, green, and blue channels, and the selected green channel with optimized color balance was saved as a separate file. Then, MRI Adipocyte Tools was added to the ImageJ launcher window, and each image was analyzed under the “S” button using a simple segmentation algorithm and binary watershed. The thresholding method was named “triangle” with minimum and maximum sizes set between 4000 and 400,000. Cells that could not be recognized by the program were added by freehand tracing. Finally, the number and mean cross-sectional area of adipocytes were calculated. Distribution graphs were plotted against cross-sectional area as the parameter with 10 specified bins.

### Real-time PCR

Expression level of adipose tissue markers and mitochondrial DNA (mtDNA) copy number in adipose tissue were determined by real-time PCR [14, 20].

### Western immunoblotting

Expression level of PHB and OPA1 in adipose tissue was determined by Western immunoblotting [14]. In brief, the total tissue lysates containing equal amounts of proteins (~ 20 μg/lane) were separated by SDS-PAGE and subsequently analyzed by immunoblotting using rabbit protein-specific primary antibodies (Abcam and Fisher Scientific, USA) and HRP-conjugated secondary antibody as described before. Finally, immunodetection was performed using Enhanced Chemiluminiscence kit (GE Healthcare, Mississauga, ON, Canada).

### Enzyme-linked immunosorbent assay (ELISA)

Serum adiponectin was measured using mouse adiponectin ELISA kit (R & D, USA) as per the manufacturer’s protocol.

### Glucose tolerance test (GTT) and insulin tolerance test (ITT)

GTT and ITT were performed 3 months post-surgery at 6 months of age as described previously [21, 22]. In brief, mice were fasted overnight for 16 h in GTT and 5 h in ITT. Fasting blood was collected and glucose levels were taken right before each test as time 0. GTT was performed with an intraperitoneal injection of glucose (1 g/kg body weight) solution in saline, and ITT was performed with an intraperitoneal injection of insulin (0.75 U/kg body weight). Blood glucose level was measured through saphenous vein puncture at 15, 30, 60, and 120 min after glucose or insulin injection. Blood glucose concentrations were measured using an ONETouch Ultra glucometer.

### Cell isolation and primary cell culture

Visceral white adipose tissue (VAT) from the epididymal fat depot and subcutaneous white adipose tissue (SAT) from the femoral region were isolated from male and female Mito-Ob and wild-type mice between 3 and 4 months of age. In brief, tissues were minced and homogenized through enzymatic digestion by collagenase I in HEPES buffer at 2–4 g tissue samples per 5 ml working solution. The digesting mixture was incubated in a shaking water bath at 37 °C for 30 min with periodic vortexing until the mixture exhibited a creamy consistency. It was passed through a 250-μm nylon mesh filter and centrifuged at 1000×*g* for 5 min. Pellets were washed with Dulbecco’s modified Eagle’s medium (DMEM) and centrifuged again at 1050×*g* for 5 min. Cell pellets were re- suspended in DMEM and cells plated on 30-mm petri dishes. Cells were then monitored every other day for growth. Upon confluency, cells were passaged into separate plates in equal numbers for subsequent treatments. Preadipocytes were passaged only once in the study. Primary cultures were maintained in DMEM containing 10% FBS, and 5% penicillin-streptomycin in a humidified incubator at 37 °C and 5% CO_2_. Cells were seeded at a density of 3 × 10^5^ measured by a TC20 Automated Cell Counter (Bio-Rad, USA). One day after confluency, cells were incubated in differentiation media containing 40 μg/mL 3-isobutyl-1-methylxanthine (IBMX), 400 ng/mL dexamethasone, and 0.5 μg/mL insulin in DMEM [11, 23]. Two days after induction, cells were switched to maintenance medium containing 1.0 μg/mL insulin in DMEM for the rest of the differentiation process. Medium was changed every 2 days. 17β-estradiol (E2) and testosterone (T) stocks (1.0 μM) were prepared by dissolving crude powder with absolute ethanol and subsequently diluted to the working concentrations of 10 nM and 100 nM, respectively before treatment [24]. Sex steroids were added with differentiation media on day 0.

### Oil Red O staining

Primary adipocytes were fixed on days 0, 4, 8, and 12 in 10% formalin in PBS at room temperature for 1 h. After washing thoroughly with ddH_2_O and dehydrated in 60% isopropanol, cells were incubated with a working solution of Oil Red O dye for 2 h. Excess Oil Red O staining was removed and cells were washed immediately with sufficient amount of ddH_2_O. Photomicrographs of Oil Red O stained lipid droplets in adipocytes were captured using Olympus BX40 microscope and Lumenera Infinity software (Lumenera Corporation, Ottawa, ON, Canada). The dye was then eluted by adding 100% isopropanol and quantified through absorbance measurement of OD at 500 nm using 100% isopropanol as a blank.

### Statistical analysis

All data were analyzed using Student’s *t* test or two-way ANOVA with Dunnett’s multiple comparison. Age- and sex-matched wild-type and sham-operated mice were included as controls. Results were expressed as mean ± SEM. A *p* value of < 0.05 was considered statistically significant in all cases. The graphs were plotted using GraphPad Prism 6 software (La Jolla, CA, USA).

## Results

### Effects of gonadectomy on body weight of Mito-Ob mice

**Fig. 1:**
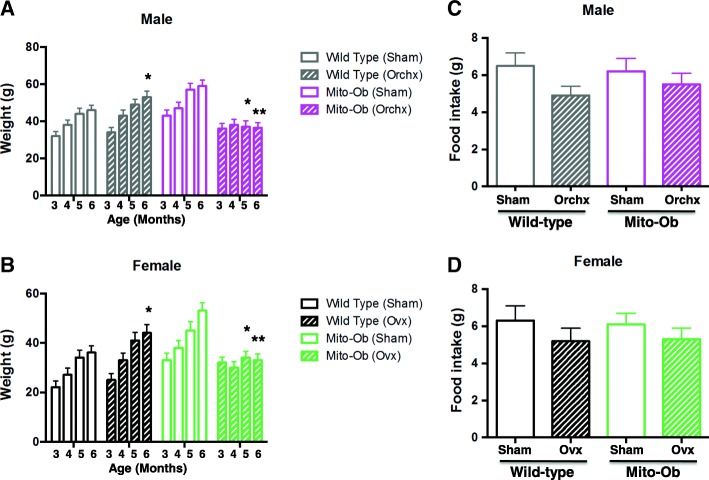
Left panel: Histograms showing the effect of gonadectomy on the body weight of **a** male Mito-Ob mice and **b** female Mito-Ob mice. Age- and sex-matched sham-operated Mito-Ob mice and wild-type mice are shown as controls. Data are presented as mean ± SEM (*n* = 8). *p < 0.05 and **p < 0.01 indicate age- and sex-matched gonadectomized mice vs sham-operated control within each experimental group. Right panel: Histograms showing the effect of gonadectomy on the food intake in **c** male Mito-Ob mice and **d** female Mito-Ob mice. Age- and sex-matched sham-operated Mito-Ob mice and wild-type mice are shown as controls. Data are presented as mean ± SEM (*n* = 6). No significant difference was found between sham-operated control and gonadectomized mice within each experimental group. Ovx—ovariectomy; Orchx—orchiectomy

Orchiectomized male Mito-Ob mice had significantly lower body weight (*p* < 0.05–0.01) 2 months post-surgery compared with sham-operated male Mito-Ob mice (Fig. 1a). In contrast, orchiectomized wild-type mice showed increasing body weight compared with sham-operated wild-type mice, which also became significantly different (*p* < 0.05) 2 months post-surgery (Fig. 1a). These data suggested that male Mito-Ob mice required testicular steroids for the development of obesity.

Consistent with previous literature, ovariectomy resulted in an increase in the body weight of wild-type mice, which became significantly higher than sham-operated control mice 2 months post-ovariectomy ((*p* < 0.05), (Fig. 1b). Similar to the male Mito-Ob mice, ovariectomized female Mito-Ob mice had significantly lower body weight (*p* < 0.05–0.01) 2 months after surgery compared with sham-operated control group. Collectively, these data suggested that sex-neural weight gain observed in male and female Mito-Ob mice was stopped by the absence of sex steroids.

### Effects of gonadectomy on food intake in Mito-Ob mice

To determine whether changes in the body weight of gonadectomized Mito-Ob and wild-type mice were due to corresponding changes in food consumption, food intake was monitored. No significant difference in food intake was observed between wild-type and Mito-Ob mice in sham-operated groups (Fig. 1c, d). After gonadectomy, a decreasing trend in food intake was found in both wild-type and Mito-Ob mice (Fig. 1c, d), implying regulation on satiety may be altered due to lack of sex steroids. However, there was no significant difference between the two gonadectomized groups, which supported our previous conclusion that any change in body weight was independent of food intake.

### Effects of gonadectomy on adipose tissue in Mito-Ob mice

**Fig. 2:**
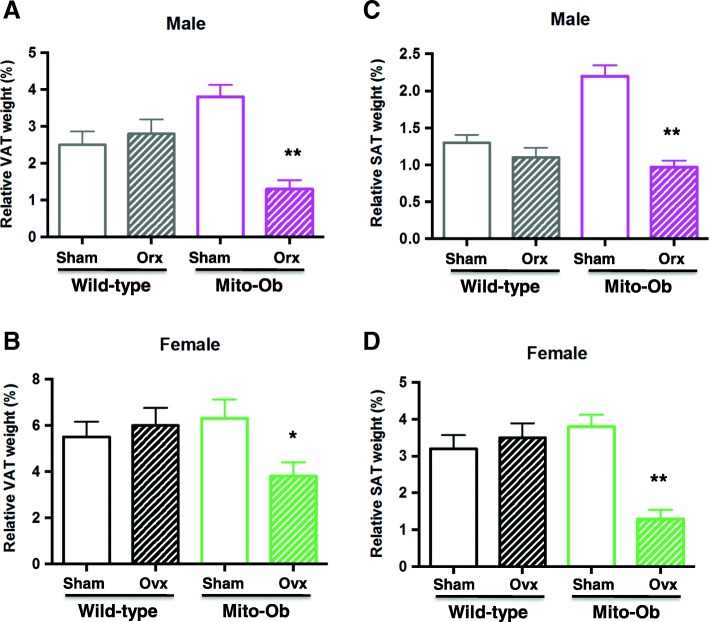
Left panel: Histograms showing the effect of gonadectomy on VAT weight in **a** male Mito-Ob mice and **b** female Mito-Ob mice. Age- and sex-matched sham-operated Mito-Ob mice and wild-type mice are shown as controls. Right panel: Histograms showing the effect of gonadectomy on SAT weight in **c** male Mito-Ob mice and **d** female Mito-Ob mice. Age- and sex-matched sham-operated Mito-Ob mice and wild type mice are shown as controls. Data are presented as Mean ± SEM (*n* = 8). *p < 0.05 and **p < 0.01 represent significant differences between age and sex match gonadectomized mice and sham-operated control within each experimental group. No significant difference was found between sham-operated and gonadectomized wild-type mice. Ovx—ovariectomy; Orx—orchiectomy; SAT—subcutaneous adipose tissue; VAT—visceral adipose tissue

To explore the effect of gonadectomy on adipose tissue, both visceral and subcutaneous fat depots were examined 3 months after gonadectomy. In males, orchiectomy resulted in significant decrease in SAT and VAT weights in Mito-Ob mice compared with sham-operated mice (*p* < 0.01, Fig. 2a, c). The relative decrease in VAT was greater compared to SAT (Fig. 2a, c). These differences were not observed between orchiectomized and sham- operated wild-type mice (Fig. 2a, c).

In the case of female mice, ovariectomized wild-type mice exhibited increasing adiposity in both depots, whereas a significant reduction in adipose tissue weight (*p* < 0.05–0.01) was seen in Mito-Ob mice compared with respective sham-operated mice (Fig. 2b, d). The relative reduction in adipose tissue weight was more pronounced in SAT compared to VAT (Fig. 2b, d). Collectively, these data suggested that PHB overexpression in adipocytes produced depot-specific effects in the presence and absence of gonadal sex steroids in male and female Mito-Ob mice.

### Histological and morphometric analyses of adipose tissue in Mito-Ob mice

**Fig. 3:**
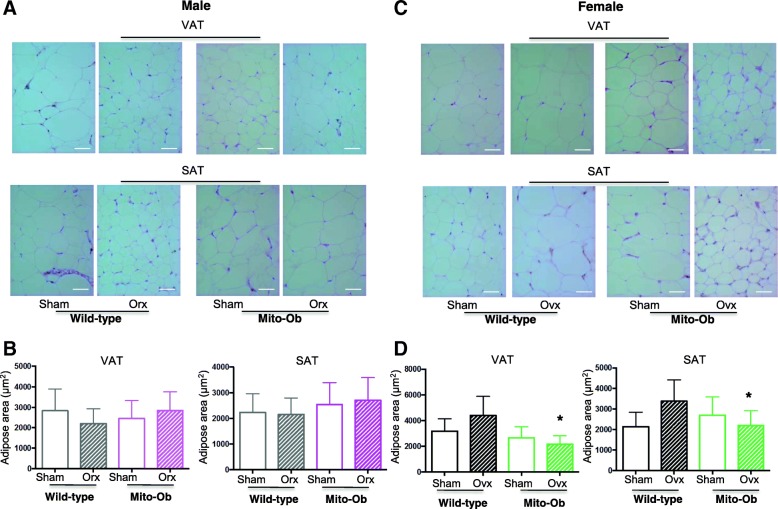
**a**, **c** Representative histomicrographs showing hematoxylin and eosin stained VAT and SAT from gonadectomized (Ovx or Orx) and sham-operated (Sham) male and female Mito-Ob and wild-type mice (40×). **b**, **d** Respective histograms showing quantification of adipocytes area. Data are presented as mean ± SEM (*n* = 8). *p < 0.05 represent significant differences between Ovx mice and sham control. No significant difference was found between sham-operated and gonadectomized male and female wild-type mice. Scale bars = 20 μm. Ovx—ovariectomy; Orx——orchiectomy; SAT—subcutaneous adipose tissue; VAT—visceral adipose tissue

Histological and morphometric analyses of adipose tissue from gonadectomized Mito-Ob mice revealed sex-specific alterations in adipocyte sizes in both SAT and VAT depots. In male Mito-Ob mice, no significant difference was found in the size of subcutaneous and visceral adipocytes between orchiectomized and sham-operated groups (Fig. 3a, b). However, adipocytes from orchiectomized wild-type mice reduced in size in VAT compared with those from sham-operated group (Fig. 3a, b).

In female Mito-Ob mice, ovariectomy resulted in a significant reduction in adipocyte size in both VAT and SAT depots compared with sham-operated mice (*p* < 0.05, Fig. 3c, d). In contrast, ovariectomy in female wild-type mice resulted in an increase in adipocyte size (Fig. 3c, d). Taken together, these data suggested that under PHB overexpression background, sex steroids played a role in regulating cellular dynamics and triglyceride homeostasis of adipocytes that is sex-dimorphic and depot-specific.

### Size frequency distribution of adipocytes in VAT

**Fig. 4:**
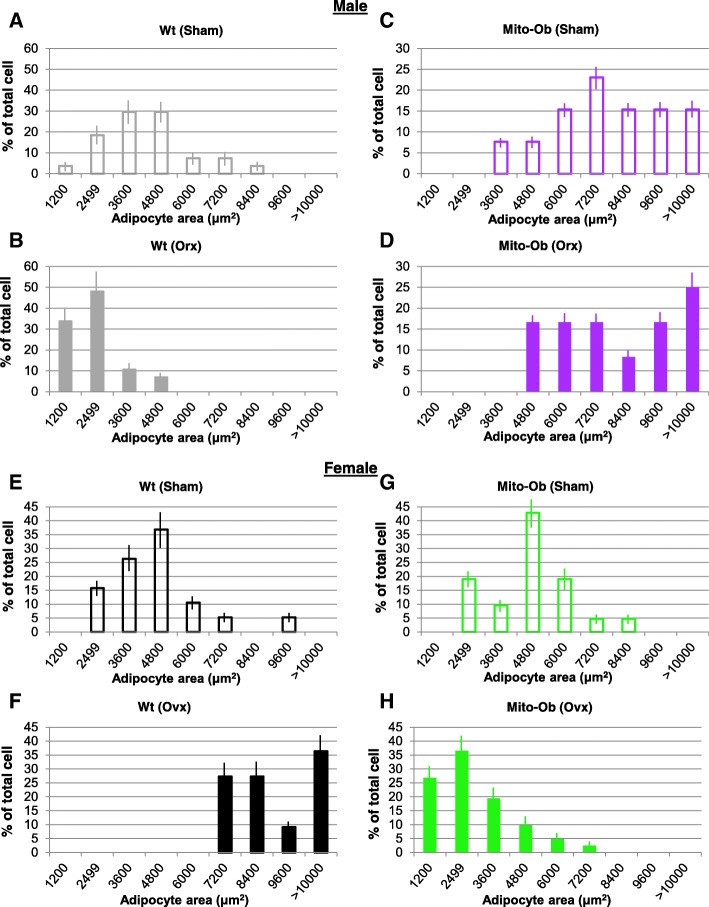
**a**–**d** Histograms showing the effect of orchiectomy (Orx) on the frequency distribution of adipocytes size in male Mito-Ob mice compared with age- and sex-matched sham-operated control. Orchiectomized and sham-operated (Sham) wild-type (Wt) mice are shown as control. **e**–**h** Histograms showing the effect of ovariectomy (Ovx) on the frequency distribution of adipocytes size in female Mito-Ob mice compared with age- and sex-matched sham-operated control. Ovariectomized and sham-operated wild-type mice are shown as control. Data are presented as percentage mean of total cells ± SEM (*n* = 8)

The frequency distribution of adipocytes showed an overlapping pattern between orchiectomized and sham- operated Mito-Ob mice (Fig. 4c, d). The cumulative numbers of larger adipocytes were higher in sham-operated Mito-Ob mice compared with the orchiectomized mice (Fig. 4c, d). However, in orchiectomized wild-type mice, the frequency distribution pattern was distinct from Mito-Ob mice, which peaked toward smaller sizes (Fig. 4a, b).

In female Mito-Ob mice, ovariectomy resulted in a shift toward smaller-sized adipocytes (Fig. 4g, h). However, an opposite trend was observed in the ovariectomized wild-type mice, where larger adipocytes were more frequently seen (Fig. 4e, f). Interestingly, opposite trends in the frequency distribution of adipocytes were observed between gonadectomized male and female Mito-Ob mice (Fig. 4d, h), which were exactly opposite to the pattern observed in gonadectomized wild-type mice (Fig. 4b, f). Collectively, these data suggested that the loss of sex steroids differentially affected size frequency distribution of adipocytes in male and female Mito-Ob mice compared with respective wild-type mice.

### Effects of gonadectomy on PHB and OPA1 expression in VAT

**Fig. 5:**
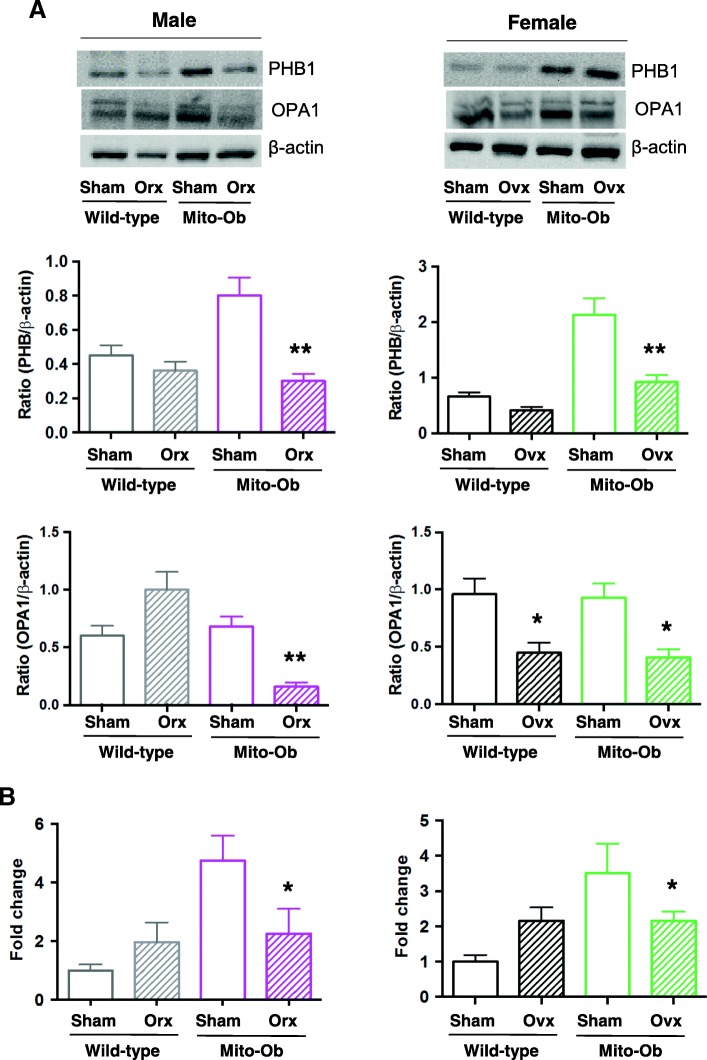
**a** Upper panel: Representative immunoblots showing PHB and OPA1 expression levels in visceral adipose tissue (VAT) from gonadectomized and sham-operated Mito-Ob mice and wild-type mice. Middle and lower panel: Histograms showing quantification of PHB and OPA1 levels. Data are presented as mean ± SEM (*n* = 4). * *p* < 0.05 represent significant differences between orchiectomized or ovariectomized Mito-Ob mice and sham control mice. **b**. Histograms showing mitochondrial DNA (mtDNA) copy number in VAT form gonadectomized Mito-Ob and wild-type. Data are presented as mean ± SEM (n = 4). * *p* < 0.05 represent significant differences between ovariectomized Mito-Ob mice and sham control mice. No significant difference was found between sham-operated and gonadectomized wild-type mice. Orc—orchiectomized; Ovx—ovariectomized

To determine whether gonadectomy alters PHB expression and mitochondrial function in adipose tissue, PHB and OPA1, a dynamin-related GTPase in the mitochondria, were measured by Western immunoblotting in VAT. A significant decrease in PHB level was found in orchiectomized Mito-Ob mice compared with sham-operated male Mito-Ob mice (Fig. 5a). A similar trend was observed between orchiectomized and sham-operated wild-type mice (Fig. 5a). In male Mito-Ob mice, a significant decrease in OPA1 level was also found in orchiectomized Mito-Ob mice compared with sham-operated control; however, the opposite trend was observed in orchiectomized wild-type mice (Fig. 5a).

Similar to male Mito-Ob mice, a significant decrease in PHB and OPA1 levels was observed in ovariectomized Mito-Ob mice compared with sham-operated control mice (Fig. 5a). A similar trend in PHB level was also found between ovariectomized and sham-operated wild-type mice (Fig. 5a); however, unlike male wild-type mice, a significant decrease in OPA1 was found in ovariectomized wild-type mice. Taken together, these data suggested a potential relationship between PHB and sex steroids in the regulation of sex differences in mitochondrial biology in VAT.

### Effects of gonadectomy on mtDNA copy number in VAT

Similar to OPA1, gonadectomy significantly reduced mtDNA copy number in VAT of male and female Mito-Ob mice compared with respective sham-operated Mito-Ob mice, whereas an opposite trend was observed between orchiectomized and sham-operated wild-type mice (Fig. 5b). Collectively, these data suggested a context-dependent role of PHB in mitochondrial biogenesis in VAT, including sex differences.

### Effects of gonadectomy on the expression of adipose tissue marker genes

**Fig. 6:**
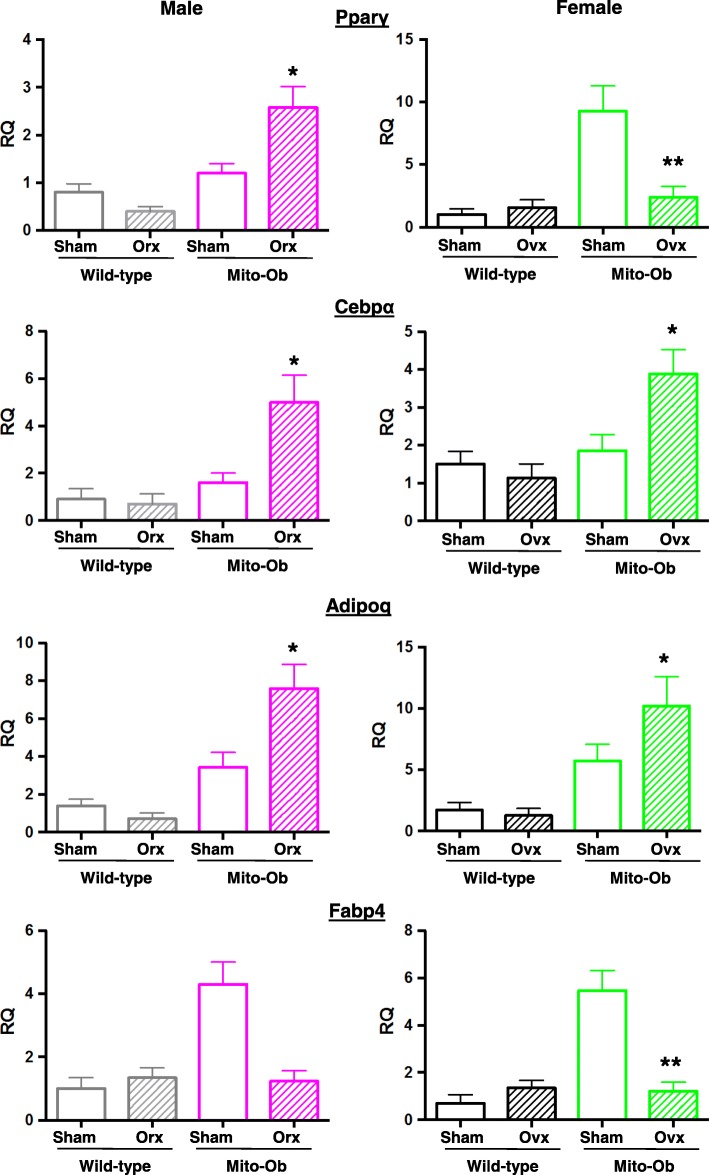
Histograms showing expression levels of adipogenic markers genes in visceral adipose tissue from gonadectomized and sham-operated Mito-Ob and wild-type mice as determined by real-time PCR. Data are presented as Mean ± SEM (*n* = 4). *p < 0.05 and **p < 0.01 represent significant differences between gonadectomized mice and sham control within genotype. RQ—relative quantification; Orx—orchiectomized; Ovx—ovariectomized; Adipoq—adiponectin

In VAT from male mice, the expression of adipocyte marker genes (*Paprγ*, *Cebpα*, and *Adipo*) were significantly increased in orchiectomized Mito-Ob mice and showed a decreasing trend in gonadectomized wild-type mice (Fig. 6). Interesting, *Fabp4* expression showed an opposite trend in gonadectomized mice compared with sham-operated control mice (Fig. 6).

In female mice, expression levels of *Cebpα* and *Adipoq* showed a pattern similar to male mice, which were significantly increased in ovariectomized Mito-Ob mice; however, *Paprγ* and *Fabp4* showed an opposite pattern, which were significantly decreased in ovariectomized Mito-Ob mice (Fig. 6), whereas an opposite trend was observed in wild-type mice (Fig. 6). Collectively, these data suggested that PHB differentially affected adipose tissue marker genes in the presence and absence of sex steroids.

### Effects of gonadectomy on serum adiponectin levels

**Fig. 7:**
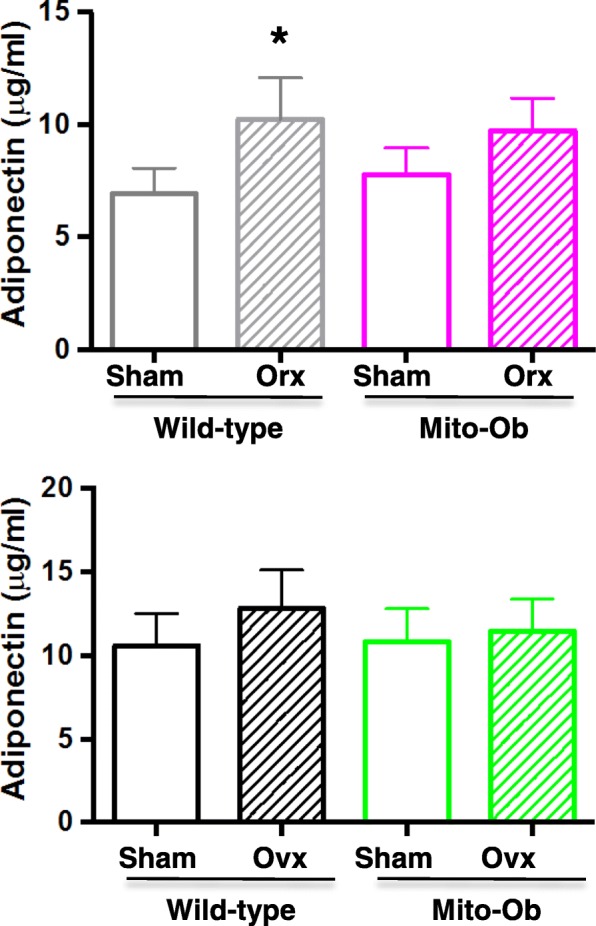
Histograms showing serum adiponectin levels in gonadectomized Mito-Ob and wild-type mice as determined by ELISA. Data are presented as mean ± SEM (n = 4). *p < 0.05 represent significant differences between orchiectomized wild-type mice and sham control mice. No significant difference was found between sham-operated and gonadectomized Mito-Ob mice and in female wild-type mice. Orc—orchiectomized; Ovx—ovariectomized

No significant change in serum adiponectin levels was found between gonadectomized Mito-Ob mice and female wild-type mice respective to their sham-operated control mice (Fig. 7). However, in orchiectomized wild-type mice, adiponectin level increased compared with sham-operated control mice (Fig. 7).

### Effect of gonadectomy on glucose homeostasis in Mito-Ob mice

**Fig. 8:**
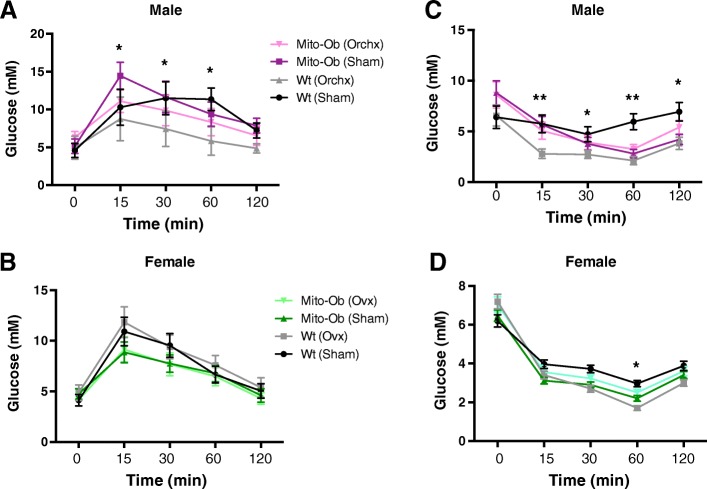
**a**, **b** Line graphs showing the effect of gonadectomy on glucose tolerance in male and female Mito-Ob mice as determined by glucose tolerance test. Sham operated mice are included as control. Data are presented as mean ± SEM (*n* = 8). *p < 0.05 represent significant differences between orchiectomized Mito-Ob mice or wild-type mice vs. respective sham-operated male mice. No significant difference was found in female mice. **c**, **d** Line graphs showing the effect of gonadectomy on insulin sensitivity in male and female Mito-Ob mice as determined by insulin tolerance test. Sham-operated mice are included as control. Data are presented as Mean ± SEM (*n* = 8). *p < 0.05 and **p < 0.01 represent significant differences between gonadectomized wild-type mice vs. sham-operated wild-type mice. No significant difference was found between gonadectomized Mito-Ob mice vs. sham-operated Mito-Ob mice. Ovx—ovariectomy; Orchx—orchiectomy; Wt—wild-type

In male Mito-Ob mice, orchiectomy significantly improved glucose disposal (*p* < 0.05) during the first 15–30 min of glucose tolerance test compared with sham-operated mice (Fig. 8a). However, this difference started to tapper after 60 min and disappeared at 120 min (Fig. 8a). An improvement in glucose disposal was also observed in the orchiectomized wild-type mice compared with sham-operated mice; the difference stayed significant (*p* < 0.05) during 30–60 min, but became insignificant at 120 min (Fig. 8a).

Interestingly, no difference in glucose disposal was found in ovariectomized female Mito-Ob mice or wild-type mice compared with respective sham control mice (Fig. 8b). However, female Mito-Ob mice displayed slightly better glucose disposal than the wild-type mice especially during the first 30 min of glucose tolerance test (Fig. 8b). Taken together, these data suggested that a sex-specific relationship exists between sex steroids and PHB in adipose tissue contribution to the regulation of glucose homeostasis.

### Effect of gonadectomy on insulin sensitivity in Mito-Ob mice

To determine whether sex steroids have a role in male-specific insulin resistance in Mito-Ob mice, we investigated the effect of gonadectomy on insulin sensitivity by insulin tolerance test (ITT). No difference in insulin tolerance was found between orchiectomized male Mito-Ob mice and sham-operated Mito-Ob mice during the first 60 min of ITT (Fig. 8c). Subsequently, insulin tolerance curve started to bifurcate between two groups but remain insignificant throughout ITT (Fig. 8c). In wild-type male mice, orchiectomy resulted in a significant increase in insulin sensitivity compared with sham-operated mice (*p* < 0.05–0.01; Fig. 8c). These data suggest that male Mito-Ob mice responded poorly to insulin compared with wild-type mice in the absence of sex steroids.

Similar to male Mito-Ob mice, ovariectomized female Mito-Ob mice displayed no difference in insulin sensitivity in comparison with sham-operated female Mito-Ob mice throughout ITT (Fig. 8d). Ovariectomized female wild-type mice showed similar but less pronounced trend in insulin sensitivity as in the case of orchiectomized male wild-type mice (Fig. 8d).

### Effect of sex steroid hormones on the differentiation of preadipocytes from Mito-Ob mice in vitro

**Fig. 9:**
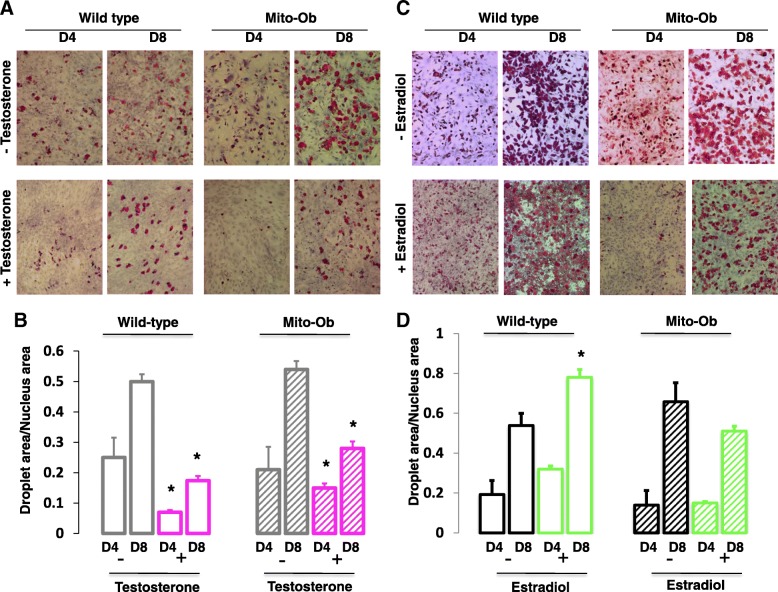
**a**, **c** Representative photomicrographs showing differentiation of subcutaneous preadipocytes from male and female Mito-Ob mice with and without respective sex steroid supplementation, as determined by Red Oil O staining (40×). **b**, **d** Respective histograms showing quantification of adipocyte differentiation. Preadipocytes from male and female wild-type mice were included as controls. Experiments were repeated for three to four times. *p < 0.05 represents significant differences between with and without sex steroid treatment within each experimental sub-groups (days, genotype and sex). D—days

To further define the relationship between PHB and sex steroids in adipocytes, we studied the differentiation of subcutaneous preadipocytes isolated from Mito-Ob mice with and without sex steroid treatments. A similar trend in the effect of testosterone was found on the differentiation of preadipocytes from wild-type and Mito-Ob mice. In both cases, testosterone inhibited preadipocyte differentiation in comparison with respective control group without testosterone supplementation (*p* < 0.05; Fig. 9a, b). No difference in adipocyte differentiation was found between wild-type and Mito-Ob in the absence of testosterone. However, in the presence of testosterone treatment, preadipocytes from Mito-Ob mice showed enhanced differentiation potential compared with preadipocytes from wild-type mice (Fig. 9a, b).

Subcutaneous preadipocytes from female wild-type and Mito-Ob mice showed similar differentiation potential without estradiol (Fig. 9c, d). However, in the presence of estradiol, an increase in preadipocyte differentiation was found from wild-type but not from Mito-Ob mice (Fig. 9c, d). Thus, a discrepancy was observed between findings from in vivo and in vitro studies on adipose tissue and preadipocytes from Mito-Ob mice indicating potential involvement of additional factors in the relationship between sex steroids and PHB in adipose tissue biology at the systemic level.

A similar trend in the differentiation potential of visceral preadipocytes from wild-type and Mito-Ob mice was observed with or without sex steroids. However, their differentiation potential in vitro was relatively poor compared with subcutaneous preadipocytes (data not shown).

## Discussion

The main findings from the effects of gonadectomy on adipose tissue biology and glucose metabolism have been summarized in Table 1. In brief, we found significant reduction in body weight in Mito-Ob mice, whereas the opposite trend was observed in wild-type mice. Intriguingly, these opposite changes occurred independent of food intake. Moreover, a depot-specific decrease in adipose tissue weight was observed in male and female Mito-Ob mice. Male Mito-Ob mice showed greater reduction in VAT whereas in female Mito-Ob mice, it was SAT. Moreover, gonadectomy differentially affected adipogenic and mitochondrial biogenesis markers in VAT. Gonadectomy improved glucose tolerance in male mice, which was more pronounced in the wild-type. Gonadectomy did not alter insulin sensitivity in male Mito-Ob mice, but it was improved in male wild-type mice. In vitro studies showed differential effects of testosterone and estradiol on the differentiation of preadipocytes from Mito-Ob mice, which were significantly inhibited by the former but not by the latter. Collectively, these findings suggested that adipose tissue from male and female Mito-Ob mice behave differently in the presence and absence of sex steroids, and consequently affected glucose homeostasis and insulin sensitivity differently.

**Table 1:** Summary of key results from gonadectomized Mito-Ob and wild-type mice relative to sham-operated control mice

Experimental groups	Wild-type		Mito-Ob	
	Sham	Gonadx	Sham	Gonadx
**♂**	- Similar food intake	- Similar food intake	- Similar food intake	- Similar food intake
		- Increased body weight		- Reduced body weight
		- No apparent change in SAT and VAT		- Reduced SAT and VAT weights
		- Reduced adipocyte size in VAT		- No significant difference in VAT adipocyte size
		- Higher cumulative number of larger adipocytes		- Higher cumulative number of smaller adipocytes
		- Improved glucose disposal		- Improved glucose disposal
♀	- Similar food intake	- Similar food intake	- Similar food intake	- Similar food intake
		- Increased body weight		- Reduced body weight
		- Increased SAT and VAT weights		- Reduced SAT and VAT weights
		- Increase in adipocyte size		- Reduced adipocyte size in both VAT and SAT
		- Higher cumulative number of larger adipocytes		- Higher cumulative number of smaller adipocytes
		- Differential effects on adipogenic and mitochondrial biogenesis markers with/without sex steroids		- Differential effects on adipogenic and mitochondrial biogenesis markers with/without sex steroids
		- No significant difference in glucose disposal		- Slightly better glucose disposal

*Gonadx* gonadectomized, *SAT* subcutaneous adipose tissue, *VAT* visceral adipose tissue

Sex steroids are known to play roles in growth and distribution of adipose tissue in the body [25], and sex differences in adipose tissue structure and function become apparent during puberty [26–28]. Our previous studies have indicated a potential link between pubertal surge in sex steroids and PHB in adipose tissue growth and metabolic regulation [14], and in this study, we found that surgical gonadectomy of Mito-Ob mice prevented weight gain in both male and female mice suggesting that PHB requires sex steroid hormones for the development of obesity in Mito-Ob mice. Thus, the effect of gonadectomy is consistent with sex-neutral obesity development in Mito-Ob, as observed previously during their phenotypic characterization [14]. Wild-type mice started to gain weight after gonadectomy, supporting previous reports that indicated the absence of gonadal estrogens or androgens led to weight gain in rodents and humans with aging [29–31]. However, this change was not found to be associated with adipose tissue area. This would imply that a relative contribution of increase in the body weight of other tissues, such as subcutaneous adipose tissue, may have added to increase in body weight. In addition, redistribution of lipid in other metabolic tissues, such as skeletal muscle and liver, may contribute to increase in body weight. Interestingly, gonadectomy-induced weight gain was not observed in Mito-Ob mice suggesting PHB overexpression in adipocytes prevented gonadectomy-induced weight gain. At the cellular level, a differential effect on adipogenic and mitochondrial biogenesis markers was also observed in gonadectomized Mito-Ob mice vs sham-operated control mice. Of note, a change in PHB protein levels was also observed in gonadectomized male and female mice compared with sham-operated control mice. Thus, it appears that PHB functions differently in adipocytes in the presence and absence of sex steroid hormones, which may involve regulation at multiple levels, such as transcription, translation, and protein stability. This may be the reason behind a discrepancy that was observed in mRNA and protein expression levels of adiponectin in gonadectomized mice. Taken together, these data indicate that PHB and sex steroid hormones may regulate each other’s function for the maintenance of adipose tissue homeostasis under normal condition. Alternatively, it is possible that PHB overexpressing adipocytes respond differently to gonadal sex steroid hormones in male and female mice as they exhibit sex differences in obesity-related metabolic phenotype [14] and associated abnormalities [18, 32, 33]. Collectively, these findings suggest that a diverse relationship exists between PHB and sex steroid hormones in the regulation of adipose tissue biology. Our findings may have implication in systemic metabolic regulation during pubertal growth, pregnancy, and aging because all these stages involve a substantial change in sex steroid levels and metabolism.

The differential effects of gonadectomy on the body weight of wild-type and Mito-Ob mice were not associated with their food consumption, which were in keeping with previous findings that Mito-Ob mice developed obesity independent of food intake [14]. Of note, an added increase in body weight of wild-type mice was observed under chow diet especially in 5- and 6-month-old mice. The exact reason(s) for this observation remain unclear; however, potential reasons may include mouse strain and diet or a combination of both. For example, in this study we have used CD-1 mice, which are known to weigh relatively more than other strain of mice. Consistent with the lack of weight gain in Mito-Ob mice, a comparative decrease in adipose tissue weight was observed in visceral and subcutaneous adipose depots. However, an associative decrease in adipocyte size was observed only in female mice. Adipocyte size varies widely depending on the amount of triglycerides stored, thus a change in frequency distribution of adipocyte size may also account for a gain or loss in adipose tissue and body weight. Analysis of frequency distribution of adipocyte size presented an increase in small adipocytes coupled with a decrease in large adipocytes only in female Mito-Ob mice. Such observations provided evidence for a role of PHB in sex differences in adipocyte frequency distributions and raised the possibility that there may be additional changes in adipocyte dynamics in response to gonadectomy in the adipose tissue of male and female Mito-Ob mice. Thus, a possibility exists that a change in the weight in other metabolic target tissues such as skeletal muscle and liver may also have contributed to the overall change in the body weight.

As mentioned previously, Mito-Ob mice develop obesity-related impaired glucose homeostasis and insulin sensitivity in a male-specific manner suggesting a potential involvement of gonadal sex steroid hormones in the observed differences in metabolic phenotype. Consistent with this notion, gonadectomy in male wild-type significantly improved glucose disposal compared with sham-operated mice. Surprisingly, a differential effect of gonadectomy was observed on glucose disposal in male and female Mito-Ob mice despite a similar reduction in adipose tissue weight and body weight. This discrepancy in glucose homeostasis would mean that PHB overexpressing adipose tissues function differently in male and female in the presence and absence of gonadal sex steroids, which may include differential production of various adipokines and metabolites. For example, ovariectomy in Mito-Ob mice (this study) and in mutant Mito-Ob mice overexpressing Y114F-PHB [32] differentially altered adiponectin levels compared with sham-operated mice. Alternatively, it may be directly due to sex-specific changes in the insulin sensitivity of other metabolic target tissues such as skeletal muscle and liver.

## Conclusion

In summary, this study revealed a sex-specific relationship between PHB and sex steroids in adipose tissue biology and metabolic homeostasis. It is anticipated that a sex-specific analysis of the underlying mechanisms involved in the interplay between PHB and sex steroids will help better define their roles in adipose tissue biology and in metabolic homeostasis. Particularly, it would be interesting to know whether mitochondrial attribute of PHB has a role in it, as mitochondria play a central role in adipocyte biology and estrogens and androgens are known to differentially affect mitochondrial biology in other cell or tissue types.

## Abbreviations

aP2: Adipocyte protein-2
E2: 17β-estradiol
Fabp4: Fatty acid-binding protein-4
GTT: Glucose tolerance test
ITT: Insulin tolerance test
Mito-Ob: An obese mouse model developed by prohibitin-induced mitochondrial remodeling in adipocytes
Orx: Orchiectomy
Ovx: Ovariectomy
PHB: Prohibitin
SAT: Subcutaneous white adipose tissue
T: Testosterone
VAT: Visceral white adipose tissue
